# Technological Advancements of Insoluble Dietary Fiber from Food By-Product Processing: A Review

**DOI:** 10.3390/foods14101822

**Published:** 2025-05-21

**Authors:** Domenico Mammolenti, Francesca Romana Lupi, Noemi Baldino, Domenico Gabriele

**Affiliations:** Department of Information, Modeling, Electronics and System Engineering (D.I.M.E.S.), University of Calabria, Via P. Bucci, Cubo 39C, 87036 Rende, Italy; domenico.mammolenti@unical.it (D.M.); noemi.baldino@unical.it (N.B.)

**Keywords:** non-starch polysaccharides, food processing, homogenization, rheology, food design

## Abstract

Insoluble dietary fibers (IDFs) represent one of the most promising candidates for novel food formulations, since they can be produced from a wide range of food by-products and wastes, have health benefits, and often enhance the rheology and stability of foods. Recently, the most innovative engineering and processing aspects of these attractive ingredients have received considerable attention. The present work is aimed at enlightening the technological state of the art regarding IDFs (much less investigated than soluble fibers, as discussed in this review). The review begins with a brief but crucial discussion on the definition of this type of dietary fiber by highlighting the raw materials, functional properties, physiological activity, and stabilization capacity in food products. The analysis of the rheological methods dedicated to the technical investigations of these ingredients and recent advancements are discussed. Finally, food processing technologies used in the formulation of foods containing insoluble IDFs, such as homogenization techniques, are discussed.

## 1. Introduction

A particular category of bioactive edible polymers, which has received special attention in recent years, for its impact on human nutrition, is that of dietary fibers (DFs) [1]. DFs include a large number of biopolymers, not only polysaccharides, with different molecular and supramolecular structures; thus, they exhibit a wide and diversified spectrum of physiological and nutritional features [1]. It is rather well-known that a proper intake of dietary fiber is associated with positive effects on human nutrition and health, contributing to mitigating the risk of several metabolic diseases, and a number of papers are available in the literature on this topic [2,3]. From a compositional point of view, DFs are composed of a water-soluble part and a water-insoluble part; therefore, dietary fibers are characterized by two specific fractions, that is, the soluble dietary fiber fraction (or “percentage” SDF_%_) and IDF_%_, i.e., IDF percentage, yielding, as a final result, the total dietary fiber fraction (TDF_%_) [4]. The soluble dietary fiber fraction (SDF_%_) is usually recognized as fermentable, whereas the insoluble dietary fiber fraction (IDF_%_) is typically non-fermentable or least-fermentable [2].

Besides the improvement in the nutritional profile, DFs incorporated into foodstuffs may act as texturing agents or, more properly, as rheological modifiers; for instance, as thickening and/or gelling components, DFs induce a significant enhancement in the consistency and stability of the final products [5,6].

The most attractive raw materials from which DFs can be obtained are typically by-products of the food processing industry [5,7]. The recovery of functional food components, such as DFs, from food by-products makes a far-from-negligible contribution to the reduction in food waste, involving economic, environmental, and ethical upgrading for the food industry worldwide [7]. Dietary fiber incorporation into foods is regulated in the European Union by EU Regulation No. 1129/2011 [8] and in the USA by the Code of Federal Regulation Tile 21 parts 172 and 182 [9]. According to the EU regulation, some dietary fibers like pectin, guar gum, and xanthan gum can be considered additives in most food preparations and needs to be indicate with an “E number” [8]; other dietary fibers like wheat fibers, apple fibers, and citrus fiber can be considered ingredients and they can simply be declared as “fiber” on the nutrition information [8], promoting the application of the claim “clean label” [10] to food products. Typically, DFs designed with an E number, such as pectin and gums, are rich in SDF_%_ (SDF_%_/IDF_%_ > 1); therefore, they are named soluble dietary fibers (SDFs). On the contrary, DFs that are simply indicated as fibers, such as citrus fiber, pea fiber, and wheat fiber, are typically rich in IDF_%_ (SDF_%_/IDF_%_ < 1), with some exceptions such as psyllium and inulin, which are almost entirely constituted by SDF_%_, and are named insoluble dietary fibers (IDFs). Food with a high content of DFs as ingredients, i.e., with IDFs, inulin, or psyllium, can also be claimed as “source of fiber” or “rich in fiber” [8].

Regardless of the type of fiber, i.e., whether SDFs or IDFs, there are some interesting studies and reviews that report the recent advances from a healthy standpoint [1,2,3,11]. Although the source search, extraction, application, and modification methods of DFs are relatively new topics, under investigation in the scientific literature, a few thematic reviews are already available [5,7,12,13]. In particular, the extraction and isolation of some dietary fibers is an attractive topic, due to both the need to obtain fibers from new sources [14] and the complexity in the isolation of some specific fibers with particular properties [15,16,17]. It is worth mentioning that, recently, the technological aspects, such as the processing, rheological, and textural properties of SDFs (like pectin and gums), have been reviewed in detail [6]. On the contrary, as far as IDFs, such as citrus fiber, wheat fiber, and pea fiber, are concerned, a lack of review work on technological advancements is still present in the literature, although there are a large number of research articles.

The present study aims to summarize the most salient technological aspects of IDFs and IDF-based food systems. Specifically, after a general contextualization and definition of IDFs and their composition, source, and role in foods and nutrition, an engineering analysis was proposed. In particular, the work is focused on the one hand on the rheological modification induced by the incorporation of IDFs into foods and on the methods used to study them and, on the other hand, on the processing used in the manufacturing of IDF-based food systems or to physically modify IDFs.

## 2. Definition of Insoluble Dietary Fibers (IDFs): Regulatory and Scientific Background

The term “dietary fiber” was used first by Hipsley [18] to describe polymeric carbohydrates of the plant cell walls resistant to digestion; this definition received a lot of attention in the following years when it was expanded to include polysaccharides not only coming from cell walls, such as pectin and gums [19]. Despite the concern of both the scientific community and regulatory authorities throughout the decades, the concept of dietary fiber is still an object of critical reflection [19].

It is worth noting that it is not easy to define what “dietary fiber” (DF) is because it is not a simple chemical compound, but a combination of different chemical substances [1]. After a long debate, the “Codex Committee on nutrition and foods for special dietary uses” proposed, in 2009, a comprehensive definition to promote international harmonization for food labelling and to recognize that the definition of “dietary fiber” can be used, independently of the way in which fiber is produced, if physiological benefits are involved [19]. In this definition, the national authorities are given the chance to include carbohydrates from three to nine monomeric units in the fiber definition, and the fractions of lignin and/or other compounds associated with polysaccharides are also considered dietary fiber (footnote of the regulatory of Codex Alimentarius). Other relevant definitions of dietary fibers, more chemically or physiologically detailed, were provided by the European Food Safety Authority (EFSA) and the American Association of Cereal Chemists (AACC), respectively [20,21]. Although the technological aspects of DFs are connected to SDF_%_/IDF_%,_ looking at all the definitions of the international authorities, it is possible to see that the distinction between soluble and insoluble fractions of DFs is not considered, which can depend on the method adopted for the determination, and they seem not to be strictly related to physiological effects. In light of all these considerations, generally speaking, insoluble dietary fibers (IDFs) can be considered a food ingredient belonging to the dietary fiber category [20,21,22], characterized by the typical health benefits of IDF_%_, but, also, to a lesser extent, by that of SDF_%_, and are often obtained from the revaluation of food processing by-products. In addition, as mentioned above, their usage also promotes the claims “clean label”, “source of fiber”, and “rich in fiber” [8,10].

## 3. Source, Composition, and Functionalities of IDFs

Considering the scientific literature, it is interesting to observe how fruit, vegetable, and cereal by-products contain a higher value of IDF_%_ than their respective unprocessed sources [23]. Relevant food processing by-products are peels and pulps from tropical fruits and citrus, tomato and apple pomaces, bran of cereals, and legume hulls, because of their high content of IDF_%_ and their commercial volumes [12]. The high content of IDF_%_ in food processing by-products is essentially due to the presence of a large content of polysaccharides, mainly cellulose and hemicellulose, that remain in the matrix after the main processing (juice and oil extraction or pectin and flour production). The main fruit, vegetables, cereal, and legume food processing by-products suitable for IDFs production, i.e., those available on the market due to the large-scale production, and their fractions of soluble (SDF_%_) and insoluble fiber (IDF_%_) are reported in Table 1. Looking at Table 1, it is interesting to note how the study concerning the extraction techniques, the chemical and functional characterization of IDFs from different commercially relevant sources such as some fruits and citrus processing by-products, were carried out during the first decades of 2000. However, other food by-products, such as legume hulls, received attention more recently [14], indicating that, although deeply investigated and reviewed [16,23], the extraction and chemical and functional characterization of IDFs from new food by-products is still an active and attractive research field.

Furthermore, considering Table 1, it is easy to see that, even though the content of IDF_%_ in the plant by-products was higher than that of SDF_%_, as previously discussed, IDFs still contained a non-neglectable fraction of soluble biopolymers. 

From Table 1, it is possible to observe that, although IDFs have been extracted and studied from a variety of sources, the greater number of scientific papers are focused on two macro-categories of sources: citrus and cereal. In particular, these two categories of fiber are the most investigated and used in industry, as suggested by the large number of research papers discussing their use and by the large number of commercial products containing them.

From a compositional perspective, IDFs are a complex mixture of macromolecules belonging to the following groups (ordered by relevance): insoluble polysaccharides such as cellulose and hemicellulose; soluble polysaccharides such as pectin, β-glucans, and lignin (anchored to the insoluble polysaccharides); and even traces of bioactive compounds [2].

Concerning the physiological activity of IDFs, the prevalent component is IDF_%_, but it can also promote secondary effects liked to SDF_%_ or even to active compounds, such as polyphenol residues in the polymer matrix [5]. The IDF_%_/ SDF_%_ ratio is important for both nutritional and technological properties. As suggested in the literature, to ensure balanced nutritional benefits from both fractions, IDF_%_/SDF_%_ should be in the range of 3:1 to 2:1 [11].

It is quite difficult to predict or identify unambiguous correlations between fiber intake and its health effects because the medical benefits are linked to their complex molecular and supramolecular structure; in fact, as already reported, fibers consist of a mixture of molecules extracted from different botanical sources [1]. Despite these limitations, different simple parameters have been developed with the aim of quantify some physicochemical behaviors indirectly related to physiological functions [23]. These parameters are commonly known as functional properties. The main functional properties are water-holding capacity (WHC), water-swelling capacity (WSC), and oil-holding capacity (OHC) [23]. The typical values of WHC and WSC are reported in Table 1, for different categories of IDFs. IDFs can exhibit a quite diversified range of functional properties depending on the kind of vegetable source and the processing they undergo; indeed, for citrus fiber, the WHC ranges from around 1.6 to almost 13 mL/g, whereas, for cereal by-products, it ranges from 3 to 3.8 mL/g [23].

Looking at the research works in the field of IDFs, it is possible to find additional functional properties related to the hydration capacity of DFs, such as the water solubility index (WSI) and bulk density (BD), but the parameters described above (WHC, WSC, and OHC), in agreement with what is reported in the literature, seem to be essential [23]. However, paying attention to the research works reported in the scientific literature, it is worth noting that IDFs are marked with further functional properties more related to their effect on human health and on physiological mechanisms [3]. Among these, the glucose adsorption capacity (GAC) and cholesterol adsorption capacity (CAC) deserve mention because of their relevance and effectiveness in mimicking the relative physiological phenomena in vitro [3]. Additionally, some authors have reported the functional properties of DFs, such as their gel-forming capacity and viscosity [23]. Nevertheless, aware of the importance of these properties from a physiological point of view and taking into account the scope of this work, viscosifying and gelling abilities are considered technological properties rather than functional properties, given their key role in food design and manufacturing.

## 4. IDFs as Rheological Modifiers in Food Systems

**Figure 1:**
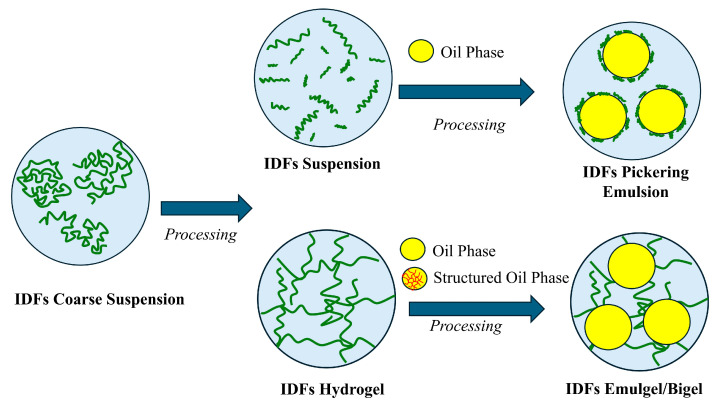
Structuring/stabilization action of IDFs in O/W systems.

Despite the differences in composition, from a physical point of view, IDF aqueous systems can be conceptualized as complex heterogeneous mixtures, that is, a suspension of solid hydrated particles dispersed in a continuous matrix buildup of a diluted polymer and/or oligomer solution, where a network of particles and polymer entanglements are also present. Generally, IDFs in water can generate different types of IDF-based systems, such as suspensions or particle hydrogels, as shown in Figure 1, depending on the particle size, composition, amount, and processing conditions [34,35,36]. In multiphasic IDF-based systems such as O/W systems, if the continuous phase is a particle hydrogel, IDFs can generate structured emulsions such as emulgels (hydrogel containing oil droplets) or bigels (hydrogel containing droplets of structured oil, i.e., an oleogel or organogel) [37,38], or a Pickering emulsion, if the dispersed phase is composed of oil droplets surrounded by small particles adsorbed at the interface [39,40]. In any case, IDFs act as rheological modifiers, firstly by forming a gel network, whereas, secondly, by surrounding the droplets, making the systems thicker (Figure 1). It is worth saying that, recently, even the role of the IDF in IDF-based systems closer to commercial food products such as yogurts or ketchup is receiving attention in the scientific literature [41,42,43].

The reduction in droplet mobility due to structuring and/or the interfacial action due to the Pickering effect involved in the incorporation of IDFs significantly contributes to the improvement in the physical stability of food [6,44]. The rheological properties of such complex heterogeneous systems are severely influenced by a wide range of peculiar factors, such as the amount and size of the hydrated dispersed particles [34], the deformability of the particles [45], and the raw material from which fiber are extracted [7,12]. In multiphasic systems, the rheology of the whole system is also dependent on the oil phase amount, droplet size and distribution, oil phase rheology, and interfacial properties [38,44,46].

Beyond the classical parameters that typically affect the rheology of heterogeneous materials, that is, the shear rate and temperature, all the aforementioned parameters should be considered when describing IDFs as rheological modifiers [45].

In this section, after a brief discussion on the stability action due to the rheological modification induced by IDFs incorporation, a basic discussion on rheological measurements and modeling, typically used for the description of food suspensions, hydrogels, and structured emulsions containing IDFs, is proposed. Finally, some significant examples of studies on the viscosity and gelling ability of IDFs are briefly discussed.

### 4.1. Stability of Food Systems Structured with IDFs

Since IDFs act as rheological modifiers, their inclusion in foods such as creams, dressings, mayonnaise, or other multiphasic food systems allows an increase in structuring and a consequent improvement in the physical stability of the product [7,47].

Typically, the methods mainly employed for studying the stability of multiphase food systems range from direct to indirect. For instance, methods ranging from visual assessments at the macroscopic scale, to the evaluation of the surface/interface interactions, to the changes in droplet size with time, passing from rheological investigations and morphological observations [46], can be employed in the study of the stability of emulsion-type systems.

To date, scientific research aimed at verifying the suitability of IDFs in enhancing the stability of multiphase food systems has taken advantage of a very large number of characterization techniques and parameters; therefore, it is arduous to summarize them in detail singularly.

In addition, the stabilization ability of IDFs depends on the dispersed phase volume fraction, pH, temperature, and salts, as well as the fiber concentration and size [39,48]. To summarize the most advanced parameters and techniques used for stability assessments of multiphase systems stabilized by IDFs, a guide is provided in Table 2.

### 4.2. Investigation Tools in the Rheology of Food Systems Structured with IDFs

The study of rheological properties in IDF-based food systems is important for tuning their texture and consistency. As highlighted by Moelants et al. [45], rheological tests and modeling represent a successful way to determine the connection between the process, structure, and functionality in IDF-based food systems. The rheological investigation typically adopted to describe the flow properties of such materials is the steady-state flow test [51] whereas, for the viscoelasticity study, dynamic oscillatory shear measurements typically in the small-amplitude regime are used. However, other types of tests are also suitable for particular investigations.

The most suitable type of instruments for the rheological characterization of IDF-based systems are rotational rheometers, which are typically equipped with parallel plates (both serrated or smooth) or a cylindrical geometry, or with special geometries, such as vanes, to overcome the practical measurement limits connected to the intrinsic issues of some samples.

Rheological models are widely used to study food systems structured with IDFs. It was found that several suspensions and hydrogels based on plant tissues, agricultural wastes, by-products [45,52], or more complex food systems, such as Pickering emulsions [39,48] and yogurts [41,42], typically exhibit a shear-thinning flow behavior that can be described by the power law model, Equation (1), or by the Herschel–Bulkley model [45,53], Equation (2), although other equations were used sporadically to describe viscosity [53]:

(1) $$\tau =K\cdot {\dot{\gamma }}^{n}$$

(2) $$\tau =\tau_{0}+K\cdot {\dot{\gamma }}^{n}$$

where K (Pa s^n^), n (−), and $\tau_{0}$ (Pa) are consistency coefficient, flow index, and yield stress, respectively [51]. Concerning the viscoelastic properties, the rheological characteristics of IDF-based food systems, such as suspensions, hydrogels, Pickering emulsions, emulgels, and bigels, can be related to the microstructure of the network through weak gel model, Equation (3) [54,55]:

(3) $$G^{*}=A\cdot \omega^{\frac{1}{z}}$$

where A (Pa s^z^) and z (-) represent the network strength and the number of rheological units interacting within, respectively [55]. In addition, the viscoelasticity of hydrogels and suspensions structured with IDFs from citrus were recently modeled by a microrheological model already adopted for colloidal gels, assuming a fractal microstructure [35,56]:

(4) $$G^{\prime }=\lambda \cdot \;\phi^{\frac{1}{3-D}}$$

where λ is a constant (Pa) related to the interactions between primary fractal flocs, ϕ (dimensionless) is the particle volume fraction, and D (dimensionless) is the fractal dimension [35]. Recently, multiphase systems containing IDFs were modeled from a rheological point of view. Specifically, emulgels and bigels structured with citrus fibers were modeled [37,38] using equations derived from the Palierne theory of composite gels by linking the complex viscoelastic modulus ($G^{*})$ to the composition and microstructure.

### 4.3. Viscosifying Ability of IDFs

The viscosity improvements induced by the incorporation of IDFs into food depend mainly on the fiber type, concentration, processing technology, and conditions. The flow properties of several suspensions of vegetable pulp and dried fibers were studied using cylindrical geometries. The results showed a higher flow index (n) and a lower consistency coefficient (K) for dried fibers than for vegetable pulps. Furthermore, K increased with concentration, while n decreased [57]. Moelants et al. [58] studied the flow behavior of carrot pulp suspensions using a six-bladed vane and found that both viscosity and viscoelasticity increase with increasing fiber concentration. They proposed a power-law-type relation (Equation (5)) for the dependence of yield stress on the pulp content:

(5) $$\sigma_{0}=a\;{\left(pulp\%\right)}^{b}$$

where $\sigma_{0}$ (in Pa) is the yield stress, pulp% is the pulp percentage, and a and b are the fitting parameters. In addition to the effects of concentration, most viscosity investigations concern with changes in the flow behavior induced by processing.

Redgwell et al. [59] investigated the viscosity of citrus fiber suspensions by varying the extrusion process parameters (and fiber concentration). Flow behavior was measured using Cuvette geometry (concentric cylinders), after a pre-shear (5 s at 100 s^−1^), by using a step rate ramp test (0.01–1 s^−1^ in 2 min, 1–100 s^−1^ in 5 min, and 300–0.02 s^−1^ in 5 min). Shear-thinning behavior was observed, and an increase in process energy led to a decrease in viscosity. Furthermore, the viscosity of the systems was correlated to the concentration of fibers through an exponential function characterized by a critical concentration value, which is strictly influenced by the process conditions, beyond which the viscosity increases sharply.

The effect of pressure, provided by high-pressure homogenization, on orange pulp suspensions (4% w/w of dried fiber) was studied by Van Buggenhout et al. [60], by measuring the viscosity using the coaxial cylinder and vane geometries and modeling them with the Herschel–Bulkley model. The authors observed an increase in the yield stress by increasing the homogenization pressure.

By studying the effect of pressure on sugar beet pulp (2 g/100 mL) through a steady-state flow test (0.1–10 s^−1^) using parallel plate geometry, an increase in apparent viscosity, which followed a power law relation was observed with increasing homogenization pressure [52]. On the contrary, the viscosity of the suspension was determined with a concentric cylinder geometry between 0–300 s^−1^ and modeled according to the Herschel–Bulkley model; a decrease in the yield stress (from 0.772 Pa to 0.125 Pa) and consistency coefficient (from 0.088 Pa s^n^ to 0.012 Pa s^n^), following the increase in homogenization pressure (0–100 MPa), was found for lily pulp suspensions [61]. The flow index increased, from 0.826 to 0.986, indicating that homogenization results in a pseudo-Newtonian behavior in the system.

Although the shear-thinning response is the most common behavior of IDF suspensions and hydrogels, some contrasting results can be found in the literature, as observed by Daou and Zhang [62]. Specifically, using a cone-plate geometry, the viscosity was measured in the shear rate range of 0–50 s^−1^ by applying a shear stress of 1–50 Pa. They tested the different fractions of fibers extracted from the defatted rice bran and observed a shear-thickening behavior for both the total, the insoluble, and the soluble fractions at two levels of concentrations, 1 and 3% w/w.

### 4.4. Gelling Ability of IDFs

As for the viscosifying effect, the gelling properties of IDFs are strictly related to the raw material from which the fiber is obtained, the concentration, and the process used to modify and incorporate it into foodstuffs. Bengtsson and Tornberg [63] investigated the impact of homogenization on the rheological properties of IDF suspensions from different sources: potato, tomato, apple, and carrot. They assessed the elastic and viscous moduli and the stress values at specific points in the linear region using vane geometry by stress sweep tests (0.01 to 100 Pa at 1 Hz). All suspensions exhibited solid-like behavior, with results showing that the rheological properties were notably influenced by both the fiber source and homogenization cycles. In particular, apple suspension showed the highest elastic modulus $G^{\prime }$, whereas carrot suspension showed the lowest; furthermore, apple and tomato fibers also exhibited a higher networking ability compared to potato and carrot fibers. The author ascribed the complex rheological outcomes to both the soluble insoluble content and particle deformability.

Willemsen et al. [64] studied the effect of residual pectin in lemon peel fibers on suspension rheology. They sequentially solubilized pectin with various solvents and removed acid, producing four suspensions (2% w/w) with varying pectin content. Frequency sweep tests (628 to 0.628 rad/s) carried out using parallel plate geometry revealed strong gel behavior with moderate stiffness in high-pectin-depleted suspensions, showing enhanced viscoelasticity compared with the typical weak gel structure.

Concerning the dependence of viscoelastic properties on the concentration, several interesting results were obtained by different authors [52,54,58], and even an empirical relation was proposed [58]. Indeed, in addition to modeling the linear viscoelastic behavior of a carrot suspension using the weak gel model, Moelants et al. [58] proposed a power law correlation (Equation (6)) to relate the effect of the pulp content on the dynamic elastic shear modulus, according to a model quite similar to the fractal one:

(6) $$G^{\prime }=c\;{\left(pulp\%\right)}^{d}\;$$

where $G^{\prime }$ (Pa) is the elastic shear modulus, pulp% is the pulp percentage, and c and d are the fitting parameters. Dynamic oscillatory shear tests have been widely used to assess the effects of processing technology and conditions on the microstructure of IDF-based systems. The effect of high-pressure homogenization on the viscoelasticity of the orange pulp suspension was investigated by measuring the dynamic moduli as a function of strain (strain sweep test, strain range: 0.1–100%, at 10 rad/s) of orange pulp suspensions using parallel plate geometry [60]. A strong correlation between the particle size and viscoelastic properties was found. The effect of process conditions on the rheological properties of citrus fiber particle gels produced via high-speed homogenization has also been extensively studied [54]. In particular, increasing the mixing intensity, in terms of power and energy, strengthened the fiber network, although very high values of energy or power did not improve the gel network. The changes in linear viscoelastic behavior due to different types of dispersion methods, that is, high-speed homogenization and microfluidization, on the linear viscoelastic behavior of citrus fiber hydrogels were also studied, reporting that both the rheology ($G^{\prime }$ and phase angle δ) and particle size are strictly dependent on the type of technique [35].

Recently, rheological tests in the linear region were also proven to be effective in the investigation of the effects of an IDF addition on the viscoelastic properties and gelation process of other food components in complex food formulations, like in the IDF addition to yogurts [41,42]. In particular, improvements in viscoelastic properties in terms of dynamic moduli $G^{\prime }$ and $G^{\prime \prime }$ and in the casein gelation mechanism can be obtained by using IDFs from different sources; however, the resulting improvements as well as the drawbacks, such as the hindrance to gelation process, were strictly related to the composition and to the preparation procedure adopted [41,42].

In addition to a rheological investigation in the linear viscoelastic region (small amplitude oscillation test (SAOS)), rheological tests in non-linear regions (large amplitude oscillatory shear (LAOS)) were used to study IDF-based suspensions. Su et al. [65] examined the viscoelasticity of citrus fiber suspensions’ viscoelasticity through LAOS tests. A parallel plate geometry was used for the tests, carried out at a frequency of 6.28 rad/s (1 Hz) and with a strain ranging from 0.1% to 1000%. Using Fourier transform and Chebyshev stress decomposition, they identified two response types (Types I and III), with the third harmonic being stronger than the fifth. Elastic behavior dominated at low strains, transitioning to a viscosity dominance at higher strains. The Chebyshev analysis showed strain-stiffening at low concentrations, with strain-softening occurring as the concentration and strain increased. Homogenization shifted the behavior from shear thinning to shear thickening. Huang et al. [52] applied a similar approach to sugar beet pulp suspensions, finding that non-homogenized samples exhibited a reduced $G^{\prime }$, whereas homogenized samples showed a strain overshoot. Lissajous curves revealed that untreated samples were elastic-dominated up to 105% strain, whereas homogenized samples became viscous-dominated at 33% strain. Additionally, processing pressures above 50 MPa shifted the behavior from a soft to hard gel, demonstrating that processing notably affects the viscoelastic properties.

## 5. Processing of Food Systems Containing IDFs

Processing methods play a crucial role in the preparation of IDF-based food systems such as suspensions, hydrogels, emulgels, and bigels, as well as in the modification of IDF properties. It is worth noting that the properties of IDFs (functional, microstructural, rheological, and nutritional) can be modified using different approaches and methods as reported by many authors [13,66,67,68]. In particular, IDF modification techniques can be classified as physical methods, such as homogenization or extrusion, chemical methods, such as alkali hydrolysis or carboxymethylation, and biological methods, such as enzymatic hydrolysis or microorganism fermentation [13,66,67,68]. According to the literature, physical modification methods, i.e., techniques in which a mechanical stress is used to change fiber properties, are the most relevant since they are cost-effective, easy to operate and to scale up, and do not generate chemical and biological wastes [13,66,67,68]. Furthermore, besides modifying IDF properties, the techniques belonging to physical methods can be effectively used for the preparation (processing) of food-type matrices containing IDFs. Because of their industrial relevance in both the modifications of IDFs and the production of IDF-based food systems, in this section, only physical methods are critically and systematically reviewed.

**Figure 2:**
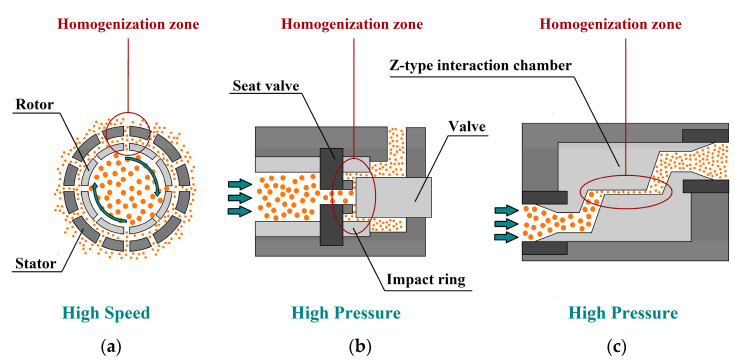
Working principle of homogenization methods in IDF processing: (**a**) high-speed homogenizer (HSH), (**b**) high-pressure homogenization (HPH), and (**c**) dynamic high-pressure microfluidizer (DHPM).

Among all the physical modification techniques, homogenization processes are the most used methods for the modification of IDFs and for the processing (preparation) of IDF-based systems [13,66,67,68]. Indeed, as it is emerged from the analysis of the literature results, high-speed homogenization (HSH), high-pressure homogenization (HPH), and dynamic high-pressure microfluidization (DHPM) appear to be the most suitable ways to effectively process IDF-based food systems and/or to modify IDFs [13,66,67,68]. The design of the basic construction features and the operating principle of the main homogenization techniques used for IDF processing are reported in Figure 2. It is worth mentioning that some of these processes can be coupled to achieve optimal preparation, generating a multistep protocol.

In addition to the aforementioned homogenizations, extrusion cooking results to be a feasible method for processing IDF-based products and/or for the physical modification IDFs [69]. Although less used than homogenization, the extrusion cooking of IDFs has received subtle attention for a long time [69]. In addition, in order to maintain an exhaustive approach, the novel but still pioneering physical modification, successfully adopted for processing IDF-based systems and/or for the physical modification of IDF properties, will be included in the present section. In the following paragraphs, only some significant recent works, considered particularly representative, will be discussed by highlighting the role of process variables on the properties of IDFs and IDF-based systems.

Examples of processing protocols that exploit one or more processing techniques used for the preparation of IDF-based food systems are reported in Table 3. From Table 3, it is easy to observe how IDF-based systems are processed using different methods, which allows the production of a wide range of final food products, some of them very close to commercial food products such as yogurts, meat emulsions, ketchup, cheese sauce, purée, acid milk, and 3D-printed cookies. It is also interesting to note that, in addition to nutritional and chemical characterizations, there is a significant tendency to use rheological and microstructural characterization in both process investigations and product design.

### 5.1. High-Speed Homogenization (HSH)

High-speed homogenization (HSH), also known as rotor–stator homogenization (RSH), is a widely used technique in food processing to disperse, emulsify, and mix food ingredients and vegetable matrices [78]. Research indicates that HSH is often employed as a pre-dispersion step in the preparation of IDF-based food systems, even if, in some cases, both the suspension-type and emulsion-type food matrices can be prepared solely by HSH, demonstrating satisfactory quality and stability (Table 3).

The effects of power and energy on the rheological and microstructural properties of citrus fiber suspensions were extensively studied by Lupi et al. [54]. They investigated the relationship between the operational parameters (speed and time) and the power of HSH using calorimetric methods. Homogenization was conducted under isoenergy (16.2 kJ/kg) and isopower (22 and 54 W/kg) conditions. Rheological and microstructural analyses showed that gel strength increased with both mixing power and energy. The extension of the network seemed largely independent of the process conditions, although a slight reduction was noted with an increasing fiber concentration. In a subsequent study by the same group a different HSH devices was used and compared to dynamic high-pressure microfluidization (DHPM), yielding consistent results and confirming the effectiveness of this techniques in dispersing IDFs [35]. 

Response surface methodology was employed to study the effects of homogenization speed and time on the chemical and physical properties of pulp from bael fruit (*Aegle marmelos* L.) [79] treated with HSH. Using parameters such as color, total soluble solids, β-carotene, ascorbic acid content, and viscosity, the optimal conditions were found to be 10,700 rpm and 43 min of homogenization. 

Wang et al. [43] prepared tomato fiber suspensions at varying concentrations (0.1–1% w/w) using HSH at speeds ranging from 3400 to 14,000 rpm for 12 min. Both HSH and high-pressure homogenization (HPH) at moderate pressure (0–10 passes and 0–10 MPa) improved the appearance and consistency of the suspensions, whereas the color parameters (L, a, and b) remained unchanged. In particular, for the highest fiber fraction (1% w/w), the samples produced by HSH and HPH showed a similar viscosity, whereas, for low fractions, the HPH allows for the formation of a more viscous suspension. In contrast, at the highest fiber fraction, the $G^{\prime }$ of the HSH-treated sample more closely resembles that of the untreated sample than that of the HPH-treated samples. Overall, the authors report that HPH was slightly more effective, leading to a tomato sauce comparable in quality to that of commercial ketchup. 

Furthermore, high-speed homogenization was found to be particularly effective also for the preparation of multiphasic systems structured with IDFs, such as Pickering emulsions, emulgels, and bigels [37,38,48], or processed cheese sauces [72].

### 5.2. High-Pressure Homogenization (HPH)

High-pressure homogenization (HPH) is a nonthermal, continuous process commonly used in the food industry for various purposes [80], including the preparation of IDF-based food systems. It is often employed alone or in combination with mechanical pretreatments, such as high-speed homogenization (HSH), to create refined products. HPH has become the primary method for preparing IDF-based foods, as demonstrated by numerous scientific studies. 

Van Buggenhout et al. [60] examined the effects of different pressures in HPH on the physical, chemical, and functional properties of orange pulp suspensions. They compared samples processed by homogenization at 200 and 800 bar and simple blending. The homogenized suspensions had a better appearance, whereas the functional properties did not change significantly compared to the blended samples. However, HPH notably affected the rheological behavior, increasing the consistency and reducing the particle size from 409 to 49 μm. J. Liu et al. [61] improved the stability of lily pulp suspensions by using HPH. After pretreatment of the pulp with a domestic blender and homogenization at various pressures (0 to 100 MPa), they observed a reduced particle size, narrower distribution, improved physical stability, and increased total soluble solids with the increase of pressure. In particular, after changing the pressure from 0 to 80 Pa, the particle mean diameter changes from 132.8 μm to 68.4 μm, whereas the total soluble solids change from 2 °Brix to 3.3 °Brix. The process also significantly decreased the yield stress and consistency coefficient, whereas the flow index increased as the pressure increased. Castro et al. [81] prepared IDF-based suspensions from yacon roots using HPH after knife milling pretreatment. The HPH process, performed at 90 bar over multiple cycles, produced stable suspensions, demonstrating the effectiveness of the process for treating different fiber sources. Suspensions made from apple, tomato, potato, and carrot fibers were also processed using HPH by Bengtsson and Tornberg [63]. These suspensions, at concentrations of 0.8% and 1.2% w/w, were pretreated with a food processor before HPH at 90 bars. The study showed that different fiber types and concentrations require varying levels of treatment to achieve the desired consistency and particle size.

Many studies highlight the need for a pretreatment step, especially when starting from solid or pulp-like raw materials, to avoid the blockage of systems and to work with more contracted matrices. High-speed homogenization (HSH) is commonly used before HPH to create treatable food matrices.

For example, Su et al. [82] prepared citrus fiber suspensions using a combination of HSH and HPH. After pretreatment with HSH for 2 min, the suspensions were further processed using HPH at pressures of 90 and 160 MPa over three cycles. Mechanical treatment improved both the microstructural and functional properties of the fibers, which were also significantly affected by the initial particle size of the fiber. In particular, for instance, in samples with small fibers (10 and 40 μm), homogenization treatment induced the larger improvement in functional properties, for instance, WHC changes from approximately 20 g/g to 40 g/g for fiber with a size of 10 μm and from 25 g/g to 40 g/g for fiber with a size of 40 μm. Although suspensions were obtained and characterized, even bypassing HSH [65], some limitations for high (3% w/w) and low (2% w/w) concentrations were observed. Lemon peel fibers obtained through various extraction methods were processed using HSH, followed by HPH [64,83]. In these studies, the suspensions were first dispersed with HSH at 8000 rpm for 10 min and then homogenized with HPH at pressures of 20 MPa and 80 MPa. The pretreatment steps, combined with HPH, significantly altered the fiber properties, including their behavior in response to pH and salt. Juric et al. [84] treated tomato peel suspensions with HSH at 20,000 rpm for 5 min and sieved them before homogenization with HPH at 100 MPa over multiple passes. This combination of mechanical treatments was successful in producing suspensions with improved physicochemical properties, leading to tomato-based products with qualities comparable to those of commercial ketchups. Sugar beet pulp suspensions were processed using HPH after pretreatment with HSH [52]. The suspensions, with a 2% w/w concentration, were homogenized at various pressures (0 to 100 MPa) for three cycles. The authors observed significant changes in the microstructural and rheological properties of the fibers as a function of pressure. Specifically, by increasing the pressure from 0 to 15 MPa, the median diameter (D_50_) increased from 43 μm to 72 μm; for a higher pressure (25–100 MPa), it decreases up to 38 μm. Concerning the flow parameters, with the increase in pressure from 0 to 100 MPa, the consistency coefficient was found to increase from 0.007 Pa s^n^ to 10.8 P s^n^, whereas the flow index decreases from 0.63 to 0.049. Similar trends were found for $G^{\prime }$ and $G^{\prime \prime }$, also modeled with power-law-type relations. Soybean hull residues were used to create IDF-based suspensions using a sequential procedure of HSH, followed by HPH [85]. After pre-homogenization, the fibers were treated with HPH at pressures ranging from 300 bar to 1000 bar over three cycles. The study found that pressure variation affected fiber properties, such as the particle size and functional attributes, leading to suspensions with distinct rheological characteristics.

Ball milling is another common pretreatment method used before HPH to reduce the size of IDF powders. Yin et al. [86] processed soybean okara fiber by ball milling before suspending it in water and treating it with HPH. Similarly, Jiang et al. [87] used ball milling to prepare citrus fibers, which were then homogenized using HPH at 60 MPa and 100 MPa over four cycles. Both studies demonstrated that the combination of ball milling and HPH improved the microstructural and functional properties of the fibers. Van Audenhove et al. [88] treated tomato fibers using cryogenic ball milling, followed by HSH and HPH. The cryogenic milling process caused a collapse of the fiber microstructure, but the HPH step restored some of the desired properties, such as the re-functionalization of the fibers. Huang et al. [52] treated sugar beet pulp fibers using two-step milling techniques. In particular, high-speed multifunction milling and multidimensional swing high-energy nano-impact milling (ZrO_2_ balls with a diameter of 6 and 10 mm, and 2:1 ratio for 5 h) were used for particle size reduction. The obtained powder was then used for suspension preparation, by pre-homogenization with HSH (12,000 rpm for 2 min) and homogenization with HPH (0–100 MPa). After the milling treatments, fiber exhibited very a small size; specifically, the cumulative diameters D_10_, D_50_, and D_90_ were 4.2, 24.9, and 77.6 μm, respectively.

Apart from hydrogels and suspensions, HPH is also a particularly suitable technique for the preparation of more complex food systems structured with IDFs such as Pickering emulsions [39] or yogurts [42,73].

### 5.3. Dynamic High-Pressure Microfluidization (DHPM)

Dynamic high-pressure microfluidization (DHPM), also known as microfluidization, is an advanced homogenization technique in which a fluid is forced through microchannels in an interaction chamber and subjected to high-speed impacts, high-frequency vibrations, cavitation, and instantaneous pressure drops [89]. This technology is emerging in the food processing industry and shows promise in modifying food macromolecules and creating fiber-based food products. Although less established than other methods, such as high-pressure homogenization (HPH) or high-shear homogenization (HSH), DHPM has shown significant potential in preparing IDF-based food systems and improving fiber functionality in food products [89].

In recent studies, citrus fiber hydrogels were prepared using DHPM, which demonstrated improved consistency and stability compared to other methods [35]. Coarse suspensions were initially processed using HSH at 500 rpm for 2 min, and then microfluidized through two Z-type microchannels at 1020 and 1700 atm. This process reduced the particle size and improved the hydrogel quality (appearance). In particular, for particle hydrogel containing 1% w/w of fiber, the surface diameter (D_3,2_) decreases from 27.7 to 19 μm, whereas the $G^{*}$ and δ remains almost the same. A comparison of DHPM with HSH alone revealed that DHPM produced samples with a lower δ, indicating better dispersion and stability. However, limited processability has been found for concentrated systems. A study by Zhu et al. [90] demonstrated that the addition of soybean oil during DHPM processing improves the physical properties of citrus fiber powder. After mixing the fibers with oil and water using HSH, the coarse emulsion was processed via DHPM at 90 and 160 MPa for two cycles. This process significantly reduced the particle size and bulk density and increased the porosity of the fibers. Additionally, DHPM preserves the flowability of the modified powder, which is essential for various food applications. Further studies, such as those conducted by Serial et al. [91], have explored the microstructure and rheological behavior of citrus fiber suspensions processed through DHPM. Using methods such as small-angle X-ray scattering (SAXS) and rheo-MRI, the authors were able to analyze how different process conditions affected the short-range order and microfibrillar network of the fiber. They found that, on one hand, DHPM influenced the fiber structure at the micrometric level, and, on the other hand, the cross-sectional size of the individual microfibrils remained intact, indicating that the integrity of the fibers was preserved even under the high pressures of microfluidization. The cooperative flow of microfibril flocs, observed during low-shear conditions, further highlighted the enhanced functional properties introduced by DHPM. Chen et al. [92] investigated the use of DHPM to improve the properties of dietary fiber derived from peaches and oats. Both the peach pomace and commercial oat fibers were dispersed in water and processed using DHPM at 120 MPa for a single cycle. The resulting freeze-dried powders exhibited a lower size, physicochemical properties, solubility, and functional characteristics compared to the native fibers. In particular, DHPM reduced the size of peach fiber by 83% (from approximately 205 μm to 35 μm) and the size of oat fiber by 33% (from approximately 111 μm to 74 μm). The WHC of peach fiber was 5 mL/g and that of oat fiber 3 mL/g; after the DHPM process, the WHC of peach fiber reached 7.7 mL/g, whereas that of oat fiber reached 6.2 mL/g. The WSC of both fibers also notably increased following DHPM; for peach fiber, the initial value was 3.3 mL/g, whereas that of oat fiber was 1.5 mL/g, and the WSC of microfluidized peach fiber was 5.1 mL/g, whereas that of oat fiber was 1.9 mL/g. This demonstrates the versatility of DHPM in modifying dietary fibers from various sources, making it more suitable for use in fiber-enriched, low-calorie food formulations.

Further studies by Morales-Medina et al. [93] examined the effect of microfluidization on pea hull fibers with varying particle sizes. To obtain the desired particle size distribution, the fibers were ground and fractionated before microfluidization. After pretreating the coarse suspensions with DHPM at pressures of 2000 bar using a Z-type interaction chamber (200 μm), to avoid blockage of channels, multiple cycles of microfluidization were carried out. In particular, DHPM were conducted through a series of two Z-type interaction chambers (200 and 100 μm) and significant improvement of the nutritional and functional properties of the fibers and of the rheological behavior were obtained. The author found that, for samples with a similar size, D_90_ close to 80 μm, increasing pressure (from 560 to 1270 bar), and a decreasing number of cycles (from 7 to 4), the yield stress increased (from 0.79 to 1.35), whereas the consistency coefficient and the flow index remained unchanged (close to 0.1 Pa s^n^ and 0.6, respectively). Concerning the viscoelastic properties, for the same samples, an increase in both $G^{\prime }$ (from 17.2 to 23.4 Pa) and $G^{\prime \prime }$ (from 2.12 to 2.80 Pa) was observed, whereas the limit strain decreased (from 0.16 to 0.04). Larger differences were found between the rheology of the samples with D_90_ close to 80 μm and the sample with D_90_ close to 60 μm. Indeed, the latter showed a higher yield stress (1.82 Pa), lower consistency coefficient (0.88) and flow index (0.43), and higher $G^{\prime }$ (91.10 Pa), $G^{\prime \prime }$ (10.50 Pa), and limit strain (0.63)

On a larger industrial scale, DHPM has been used to modify pea fibers in a continuous process, as described by He et al. [94]. Specifically, sn industry-scale microfluidizer equipped with a pre-pulverizing unit, processed high volumes of pea fiber suspensions at pressures of 60, 90, and 120 MPa. The final product, freeze-dried for analysis, showed significant improvements in morphology, microstructure, and functional properties, and a lower particle size. In particular, D_3,2_ passed from 34.5 μm (native fiber) to 19.4 μm (fiber homogenized at 120 MPa). Microfluidization reduced the crystallinity of the fibers as reported by XRD analysis (from 28.6% of native fiber to 23.10% of fiber microfluidized at 120 MPa). DHPM was also found to be very effective in enhancing the hydration properties and oil holding capacities of pea fiber. Specifically, the WSC increased from 4.4 mL/g (native fiber) to 15.5 mL/g (fiber homogenized at 120 MPa), and the WHC increased from 4.2 g/g (native fiber) to 7.9 g/g (fiber homogenized at 120 MPa), whereas the OHC increased from 3 g/g (native fiber) to 6.3 g/g (fiber homogenized at 120 MPa). This suggests that DHPM can be effectively scaled up for industrial food processing, maintaining its capacity to enhance the nutritional and functional qualities of insoluble dietary fiber.

In addition to enhancing the functional properties, DHPM has been shown to improve the adsorption capacity of rice bran insoluble dietary fibers for heavy metals [95]. They found that higher pressures (up to 150 MPa) resulted in a greater adsorption of heavy metals under physiological conditions. This indicates that microfluidization could also be useful for food safety applications, particularly for removing or reducing harmful substances from food ingredients. In addition, DHPM was also successfully employed for the preparation of more complex food-type systems containing IDFs, such as the acid-milk gel model [74].

### 5.4. Extrusion Cooking

Extrusion is a widely used technology in both polymer processing and food industry, particularly, in the latter is more commonly known as extrusion cooking [69]. This process combines mechanical energy and heat to modify the feed matrix, which enhances the properties of food products. Most of the research on extrusion cooking focuses on SDFs such as gums; nevertheless, its effects on IDFs have also been explored. Studies have shown that extrusion cooking can increase the soluble fraction and viscosity of IDFs such as citrus fibers, onion residues, and sugar beet pulp, without causing significant thermal degradation. This suggests that extrusion cooking can improve the functional properties of these fibers [69]. A key study conducted by Redgwell et al. [59] highlighted the effect of varying the specific mechanical energy (SME) and moisture content on the properties of commercial citrus fibers during extrusion. When the SME increased from 205 kJ/kg to 479 kJ/kg, both the viscosity and water retention capacity decreased. At lower moisture levels, machine blockage occurred, whereas a higher SME triggered slight Maillard reactions. Overall, the increase in SME improved the soluble fraction of citrus fibers. In particular, samples produced with SMEs of 205, 248, 201, and 255 kJ/kg showed degrees of solubilization between 8 and 13.7%, whereas samples produced with SMEs of 406, 479, 365, and 411 kJ/kg showed degrees of solubilization of 23, 33, 30, and 36%, respectively. Extrusion cooking has proven effective for processing highly concentrated IDF-based systems, resulting in products that resemble pastes or wet powders rather than suspensions, hydrogels, or structured emulsions.

Although extrusion generally increases the solubility and fermentability of IDFs, some studies have reported conflicting results. For example, research on powdered fruit peels from orange, mango, and prickly pear showed that extrusion sometimes reduced the total fiber content and soluble fraction, indicating that the vegetable source plays a significant role in the process outcomes [96]. The same authors carried out a previous study using a factorial experimental design approach with the aim of describing the effects of process parameters on the SDF_%_ and IDF_%_ of orange peel fiber concentrate [97]. A similar study on soybean hulls demonstrated that optimal conditions, with moisture at 42.58%, screw speed at 182.46 rpm, and temperature at 87.34 °C, improved the physicochemical properties and solubility of soybean hull fibers, enhancing their nutritional features [98]. In particular, in the optimum condition (determined by the response surface method), the SDF% was 11.53%, the water absorption index was 7.77 g/g, and the SME was 246.65 w/h/kg. Recently, Schmid et al. [99] found that the extrusion cooking of chokeberry pomace increased the soluble fraction, in particular, arabinose, galactose, rhamnose, and pectin, without affecting the total dietary fiber, phenolic acid, or flavonoid content. However, high SME levels negatively affected sugars and anthocyanins.

### 5.5. Novel Processing Methods

There are a lot of new and non-conventional techniques suitable for producing IDF-based systems and/or physically modifying IDFs which can be virtually used and proposed for industrial implementation, as reported by different authors [13,66,67,68]. It is worth noting that, in contrast to conventional methods, novel processing methods are mainly aimed at modifying the properties of IDFs rather than preparing food-type systems. Between the latest generation technologies, high hydrostatic pressure (HHP) in IDF processing seems to be particularly relevant and attractive, owing to its advantages [100]. In particular, operating the HHP at a pressure of 200–400 MPa for 15–30 min the SDF_%_ increased and the functional and technological properties improved for dietary fiber from lotus root residues [101], okra soybean [102], and purple flesh potato [103].

Ultrasound-assisted dispersion is a novel technique employed for processing IDFs. In particular, a comparative study between high-intensity ultrasonication and high-pressure homogenization was conducted by Wu et al. [104] with the aim of assessing the modifications induced by the processing of okara fibers and the isolation of nanocellulose. The ultrasound processes were conducted at different energy levels (400 W—15 min, 400 W—30 min, 600 W—15 min, and 600 W—30 min) and using a power density of 0.33 W/mL, whereas the high-pressure homogenization was carried out at different pressures using three cycles. For both processes, a coarse suspension (4% w/w) was previously prepared using HSH (10,000 rpm, 5 min). Commercial citrus, apple, oat, and pea fibers were used to stabilize (by Pickering effects) emulsions produced by using high-intensity ultrasound (amplitude: 116 μm, time: 150 s, energy density: 225 kJ/L, and power: 325 W) [105]; this highlights that this technique is also suitable for the preparation of biphasic systems.

A method based on cavitation jet processing was developed for the processing and modification of IDF-based food systems [106]. Suspensions with a fiber/water ratio of 1:8 were successfully produced at 25 °C and 0.01 MPa at different processing times (3, 5, 8, 10, 12, and 15 min), highlighting the ability of this technique to produce systems with small fiber particles, a higher soluble fraction, and functional properties. Specifically, the authors found that 10 min of processing produced samples with the smallest size and best properties. In particular, D_4,3_ decreased from 2.31 to 1.51 μm, SDF% increased from 3.6% to 17.6%, WHC increased from 7.14 g/g to 9.97 g/g, WSC increased from 9.37 mL/g to 13.92 mL/g, and OHC increased from 2.46 g/g to 5.04 g/g.

Recent works reported the ability of the steam explosion process to improve the quality of IDFs from different sources [107,108]. In particular, some physicochemical and functional properties of rice bran dietary fiber can be enhanced by steam explosion treatment by changing the steam pressure (0.4, 0.8, 1.2, 1.6, and 2.0 MPa) and crushing degree (60, 80, and 100 mesh) [107]. In another comparative work, the soluble fraction of coconut dietary fiber was increased (115%) and the overall quality improved by the steam explosion process; however, the authors reported and highlighted better results for fibers treated by extrusion cooking [108].

A further method recently used for the modification of DFs is the induced electric field (IEF) treatment [109]. Using an excitation voltage of 500 V, a process time of 40 min, and NaCl concentration of 0.1%, the SDF% in wheat bran fiber increased from 7.69% to 12.02%. In addition, IEF treatment increases the porosity and hydration properties, whereas the crystallinity and thermal stability slightly decrease. In general, IEF treatment also represents a promising processing/modification technique potentially suitable for industrial applications.

## 6. Conclusions and Future Perspectives

This study underscores the critical role of insoluble dietary fibers (IDFs) in promoting healthy nutrition, sustainability, and technological innovation in the formulation of foods. IDFs can be obtained from a wide range of food-processing by-products such as citrus peels, apple pomace, and wheat bran. The source and the extraction method notably affect both the functional properties and technological properties of IDFs, which consequently exhibited a wide spectrum of performances. Among all the IDFs available, citrus fiber and cereal fibers are the most studied and used.

In general, IDFs act as rheological modifiers providing consistency, structuring, and stability to foods in which they are added. Rheological investigations represent a successful way to study IDF-based food systems. Viscosity is typically determined through steady-state flow tests, although some other types of measurements are used sporadically. Viscoelasticity is largely investigated by dynamic tests in linear conditions. For special purposes, dynamic tests were also carried out in non-linear conditions.

Among all the techniques for the physical modification of IDF and its incorporation into foods, high-speed homogenization (HSH), high-pressure homogenization (HPH), dynamic high-pressure microfluidization (DHPM), and extrusion cooking are the most effective and used. Processing profoundly affects the physicochemical features, and functional and technological properties of IDFs, and their performance in food products. The adoption of multistep processing, for example, coupling a preliminary particle size reduction before the homogenization process, results in it being widely used because it yields several advantages such as final food products with improved properties (appearance, texture, and stability) and the limitation of the blockages of the processing systems. Nowadays, conventional processes, such as HSH or extrusion cooking, are subjected to optimization, and less conventional processes such dynamic high-pressure microfluidization (DHPM) can be considered under a scale-up, whereas a lot of attention is paid to the development and application of new processing methods such as high-hydrostatic-pressure and ultrasound treatments.

The results of this study suggest that the ongoing and future research on IDF application is mainly focused on the study of the rheological properties of IDF-based systems closer to commercial foods, such as yogurt, ketchup, and sauces, or on the use of advanced rheological models. Regarding the current and future research trends on new technologies for IDF processing and IDF physical modifications, it is clear that most of the activities are aimed at developing methods able to increase the soluble fraction, such as steam explosion, rather than developing processes for food production, with the exception of ultrasonic treatment and high hydrostatic pressure, which could potentially be used for food preparation. Overall, the advancement in the field of IDFs from a technological point of view provided a far from negligible contribution to the development of food products that are both nutritionally superior and environmentally sustainable, supporting the growing shift toward cleaner and more natural food ingredients.

## Figures and Tables

**Table 1:** Food by-products sources of IDFs: IDF_%,_ SDF_%_, and TDF_%_ (the slight differences between the sum of IDF% and SDF%, and the total quantity TDF% is due to experimental error), fiber fractions, water-holding capacity (WHC), and water-swelling capacity (WSC).

Food By-Products	TDF_%_	SDF_%_	IDF_%_	SDF_%_/IDF_%_ Ratio	WHC (g/g)	WSC (mL/g)	Reference
**Fruits processing**							
*Apple pomace* var. Royal Gala	78.20	14.33	63.90	1:4.5	1.62	6.59	[24]
*Apple pomace* var. Granny Smith	60.70	4.14	56.50	1:12.9	1.78	6.89	
*Apple pomace* var. Liberty	89.8	8.20	81.60	1:9.9	1.87	6.87	
Passion fruit	81.50	35.50	46.00	1:1.3	13.5	7.2	[25]
Pineapple	75.80	0.60	75.20	1:125.3	14.6	6.6	
Guava	69.10	11.10	57.70	1:5.2	10.2	1.4	
Mango	70.00	28.20	41.50	1:1.5	6.4	4.6	
*Mango peel* var. Raspuri	54.90	17.20	37.70	1:2.2	-	-	[26]
*Mango peel* var. Badami	72.50	23.00	49.50	1:2.2	-	-	
*Cactus pear peel* var. Rojo San Martìn	45.20	10.60	35.2	1:3.3	-	-	[27]
*Cactus pear peel* var. Verde Villanueva	58.1	17.2	40.9	1:2.4	-	-	
*Cactus pear peel* var. Rojo Cenizo	50.90	19.3	31.6	1:1.6	-	-	
*Cactus pear seed* var. Rojo San Martìn	93.8	2.60	91.3	1:35	-	-	
*Cactus pear seed* var. Verde Villanueva	91.2	2.7	88.5	1:33	-	-	
*Cactus pear seed* var. Rojo Cenizo	86.7	2.4	84.3	1:35	-	-	
**Vegetable processing**							
Tomato peel	86.10	14.30	71.80	1:5.0	6.86	0.11	[28]
Carrot pomace	96.20	1.4	94.70	1:67	22.9	-	[29]
Sugarcane bagasse	90.82	0.00	90.82	-	17.3	-	
**Citrus processing**							
*Orange bagasse* var. Navel	35.40	12.60	22.80	1:1.8	10.02	-	[30]
*Orange bagasse* var. Salustiana	35.90	13.00	22.90	1:1.8	10.32	-	
*Orange bagasse* var. Valencia	36.90	11.30	25.50	1:2.3	7.30	-	
Orange peel, bagasse, and seed	63.60	17.40	46.20	1:2.7	8.71	-	[31]
Orange peel	63.70	15.60	48.20	1:3.1	9.63	-	
*Lime peel* var. *C.* Aurantifolia	70.40	21.89	48.67	1:2.2	12.84	13.64	[32]
*Lime peel* var. *C.* Latifolia	66.70	20.26	46.45	1:2.3	6.96	11.34	
*Lemon peel* var. Eureka	60.10	9.20	50.90	1:5.5	1.85	7.32	[24]
*Lemon peel* vat. Fino 49	68.30	6.25	62.00	1:9.9	1.74	9.19	
*Orange peel* var. Valencia	64.30	10.28	54.00	1:5.3	1.65	6.11	
**Cereal processing**							
Wheat bran	45.50	2.80	41.7	1:14.9	-	-	[33]
Rye bran	35.80	5.30	30.50	1:5.8	-	-	
Oat bran	21.70	4.30	17.40	1:4.0	-	-	
Corn bran	96.54	4.09	92.44	1:22.6	32.8/74.3	-	[29]
Wheat bran	92.03	0.00	92.03	-	13.9	-	
Wheat straw	97.55	0.00	97.55	-	23.7	-	
Rice fiber	98.63	0.00	98.63	-	6.4	-	
Barley hulls	102.65	4.15	98.51	1:23.7	13.8	-	
Barley straw	104.41	6.84	97.57	1:14.3	21.4	-	
**Legume processing**							
Bean hull	77.35	5.56	71.79	1:12.9	3.4	-	[14]
Chickpea hull	84.18	6.50	77.61	1:11.9	3.7	-	
Cowpea hull	70.86	1.08	69.78	1:64.6	3.8	-	

**Table 2:** Parameters typically used in the assessment of physical stability of multiphase systems structured with IDFs.

Stability Parameter	Symbol	Technique Used	References
Surface diameters, over time	D_3,2_	Laser diffraction/Indirect methods	[40,48,49]
Volume diameter, over time	D_4,3_	Laser diffraction/Indirect methods	[39]
Emulsion index	EI%	Visual inspection	[40,48]
Theoretical surface coverage	C	Coupling different methods	[40,48]
ζ-potential	ζ	Electrophoretic laser Doppler	[39]
Emulsion stability index	ESI	Laser diffraction/Indirect methods	[50]
Interfacial tension	γ	Contact angle/Pendent drop	[38,49]

**Table 3:** Processing and characterizations of IDF-based foods systems.

IDFs	Food Product	Technological and Physiological Assessments	Processing Methods	Reference
Sugar beet fiber	Meat emulsion model	Viscosity, viscoelasticity, rheological modeling, texture analysis	HSH (2250 rpm, 10 s + addition of oil, 5 s)	[70]
Okara fiber	3D-printed cookies	Viscosity, viscoelasticity, texture analysis, functional properties, stability studies	Ultrasound (400–600 W, 30 min) + HPH (5000–15,000 rpm, 10 min) + 3D-printing	[71]
Sweet corn fiber	Whole sweet corn suspension	Viscosity, physical stability, phenolic compounds, in-vitro antioxidant activity	Industry-scale microfluidization (60, 09, 120 MPa)	[47]
Bamboo, acacia, potato, and citrus fiber	Processed cheese sauces	Viscosity, viscoelasticity, texture analysis	HSH (1118 g, 2 + 10 min)	[72]
Pumpkin, carrot, green pea, and zucchini purees	Fiber-enriched yogurt	Physical-chemical profile, in-vitro antioxidant activity, texture analysis	Homogenization + homogenization for yogurt production (60 °C, 50 bar)	[73]
Soybean fibers	Fiber-enriched yogurt	Viscosity, viscoelasticity, microstructure	HPH (20–22 MPs) + yogurt production	[42]
Mulberry pomace	Fiber-enriched yogurt	Physical chemical properties, viscosity, viscoelasticity, microstructure, and texture analysis	Yogurt production	[41]
Oat bran	Acid-milk gel model	Microstructure, functional properties, viscosity, texture analysis	Enzymatic reaction + extrusion cooking + microfluidization + mixing	[74]
Grape pomace fiber	Cassava–soy–grape pomace fiber composite food model	Viscosity, in vitro digestibility studies, phenolic compounds, in vitro antioxidant activity	Extrusion cooking (60–140 °C, 200 rpm)	[75]
Apple pomace fiber	Fiber-enriched yogurt	Soluble/insoluble fraction	Mixing/homogenization (conditions not specified)	[76]
Orange fiber	Ice cream	Physical and functional properties, chemical profile	Homogenization in batch processing plant (conditions not specified)	[31]
Citrus fiber	O/W food emulgel model	Viscoelasticity, interfacial properties, modeling of viscoelastic properties	HSH (5000–10,000 rpm, 180–800 s)	[38]
Citrus fiber	O/W food bigel model	Viscoelasticity, modeling of the viscoelastic properties	HSH (2000 rpm, 120–240 s) + HSH (5000 rpm, 180–360 s)	[37]
Tomato fiber	Ketchup-like tomato sauce	Viscosity, viscoelasticity	HSH (3400–14,000 rpm, 12 min), HPH (0–10 MPa, 0–10 cycles)	[43]
Tomato fiber/wheat fiber	Pimento purée	Viscosity, thixotropy, texture analysis, functional properties	HSH (1500 rpm)	[77]

## Data Availability

No new data were created or analyzed in this study. Data sharing is not applicable to this article.
